# Diagnostics and Therapeutics in Targeting HER2 Breast Cancer: A Novel Approach

**DOI:** 10.3390/ijms22116163

**Published:** 2021-06-07

**Authors:** Chris Vi, Giovanni Mandarano, Sarah Shigdar

**Affiliations:** 1 School of Medicine, Deakin University, Geelong, VIC 3220, Australia; ViCh@deakin.edu.au (C.V.); giovanni.mandarano@deakin.edu.au (G.M.); 2 Institute for Mental and Physical Health and Clinical Translation, School of Medicine, Deakin University, Geelong, VIC 3220, Australia

**Keywords:** aptamers, HER2, breast cancer, radiolabelling, radioisotopes, antibodies, nanobodies, imaging, diagnosis, cancer

## Abstract

Breast cancer is one of the most commonly occurring cancers in women globally and is the primary cause of cancer mortality in females. BC is highly heterogeneous with various phenotypic expressions. The overexpression of HER2 is responsible for 15–30% of all invasive BC and is strongly associated with malignant behaviours, poor prognosis and decline in overall survival. Molecular imaging offers advantages over conventional imaging modalities, as it provides more sensitive and specific detection of tumours, as these techniques measure the biological and physiological processes at the cellular level to visualise the disease. Early detection and diagnosis of BC is crucial to improving clinical outcomes and prognosis. While HER2-specific antibodies and nanobodies may improve the sensitivity and specificity of molecular imaging, the radioisotope conjugation process may interfere with and may compromise their binding functionalities. Aptamers are single-stranded oligonucleotides capable of targeting biomarkers with remarkable binding specificity and affinity. Aptamers can be functionalised with radioisotopes without compromising target specificity. The attachment of different radioisotopes can determine the aptamer’s functionality in the treatment of HER2(+) BC. Several HER2 aptamers and investigations of them have been described and evaluated in this paper. We also provide recommendations for future studies with HER2 aptamers to target HER2(+) BC.

## 1. Introduction

Breast cancer (BC) is a leading cause of mortality in women worldwide [1,2,3] and is highly heterogeneous with various phenotypic expressions [4]. BC can further be classified based on the expression of three important molecular receptors: estrogen receptor (ER), progesterone receptor (PR) and human epidermal growth factor receptor 2 (HER2) [4]. Molecular analysis, determined by immunohistochemistry (IHC), is used to enhance the understanding of prognosis and predict tumour behaviour to improve therapeutic treatment strategies [4,5,6]. Overexpression of HER2 is responsible for 15–30% of all invasive BC and is strongly associated with poor prognosis and decline in overall survival [7,8,9].

Early diagnosis of BC is crucial to improving clinical outcomes and prognosis. Current imaging modalities (mammography, ultrasound and magnetic resonance imaging (MRI)) are essential for detecting anatomical details and locations of tumours for cancer diagnosis; however, they do not provide important information on the molecular characteristics of such lesions [10,11,12,13]. As a result, targeted imaging using overexpressed tumour biomarkers such as HER2 is being investigated in nuclear medicine to bridge the gap between the anatomical details and molecular characteristics of BC lesions, to further improve clinical prognosis [13]. Molecular imaging targeting BC biomarkers can increase the specificity and sensitivity of early tumour detection to improve patient outcomes [14,15,16,17].

Aptamers are short, single stranded, non-coding DNA or RNA nucleotides that are capable of binding to selected targets with remarkable specificity and affinity [18,19,20,21]. Aptamers can also be functionalised via radiolabelling with radioisotopes to be used as diagnostic (γrays) and therapeutic (α and β particles) agents [22,23]. Thus, aptamers are emerging as promising targeting agents and offer advantages over antibodies in cancer research due to their ease of in vitro synthesis; smaller size; low immunogenicity; pH and temperature stability; and functionalising capabilities that do not risk losing binding specificity or affinity to the target [24,25,26]. Due to their high target specificity and tuneable binding affinities, aptamers targeting HER2 are promising agents in nuclear medicine for the early detection and diagnosis of HER2(+) BC [25,26,27,28].

## 2. HER2 Breast Cancer

HER2 is a member of the epidermal growth factor receptor (EGFR) family that has tyrosine kinase activity. The EGFR family consists of three other receptor proteins: HER1, HER3 and HER4 [29]. This HER receptor family controls and determines epithelial cell growth, differentiation and survival [30]. Unlike other members of the EGFR family which share extracellular ligand-binding domains, the HER2 receptor protein does not exhibit any identifiable binding ligand on its extracellular domain [29,31]. Dimerisation of the transmembrane receptors results in autophosphorylation of tyrosine residues within the intracellular domains of the protein, and consequently initiates two key signalling pathways involved in cancer pathogenesis [32]. The activation of the mitogen-activated protein kinase (MAPK) and the phosphatidylinositol 3 kinase-protein kinase B (PI3K-Akt) pathways due to HER2 homo- or hetero-dimerisation promotes tumour cell proliferation, differentiation, survival and migration, causing adverse pathological disease and poor prognosis [27,32,33]. Biological responses of the activated signalling pathways are precisely dependent on the ligands involved and dimers formed; thus, dysregulation of HER receptors can result in aberrant signalling, leading to fundamental biological processes to be altered [34,35,36]. Moreover, cell polarity and adhesion are specifically disrupted by HER2 activation, which can cause aberrant asymmetric cell division and subsequent overgrowth of less differentiated cells [37,38]. This process occurs through the binding of the Par6 and atypical protein kinase C (Par6-aPKC) components of the Par complex upon HER2 activation [37]. Thus, HER2(+) tumours are poorly differentiated [36,39]. Amplification of the HER2 gene, located on chromosome 17 (17q12q21), leads to the overexpression of HER2 receptors and is highly associated with increased cell proliferation, tumorigenesis and invasion, resulting in distant metastases [33,40]. Although little is known about HER2 gene amplification’s mechanism of action, mutations arising from double-stranded breaks of DNA are the likely causes of amplification of the HER2 gene, which transforms it from a proto-oncogene to an oncogene and induces tumorigenesis [40,41,42]. Furthermore, the overexpression of HER2 is not limited to BC and has also been observed in other cancers, such as oesophageal, gastric, lung, ovarian and endometrial [9,32]. Early-stage detection of BC and identification of prognostic biomarkers would enable appropriate treatment and therapeutic actions to improve clinical outcomes and survival rates [43,44].

Compared to other sub-types of BC, HER2(+) BC has been recognised as a more aggressive early-stage BC [45]. It also demonstrates greater malignancy with poor relapse-free progression and high metastasis and recurrence rates [45,46,47]. HER2 overexpression promotes early dissemination of tumour cells to secondary organs, resulting in increased risk of metastatic disease, which is responsible for the majority of cancer morbidity and mortality [48,49,50,51,52]. Moreover, current data from tumour cell kinetics and disease progression suggest that at the time of BC diagnosis, distant micrometastases may already be present due to early intravasation and extravasation of disseminated cells [51,53,54]. Micrometastases can give rise to macro-lesions at secondary sites and initiate further metastatic disease at distant organs, such as bones, the brain, the liver, lungs and the spine, which can negatively impact the patient’s quality of life and survival [52,55,56]. Micrometastasised tumour cells can enter a dormant or quiescence state and trigger the disease at a later period when additional genetic abnormalities or conditions favourable to resuming proliferation occur (disease recurrence) [49,51,53]. Therefore, early detection of metastatic disease is critical to improving patient prognosis and clinical outcomes.

## 3. Early Detection Is Critical

Early detection of BC is crucial to improving patient outcomes, and reducing recurrence and mortality rates [11,44,50,57,58]. Significantly higher survival rates are positively correlated with smaller primary tumours in patients at the time of diagnosis [59,60]. Early detection coupled with effective treatment—surgery, radiation therapy (RT), chemotherapy, endocrine therapy (ET) or targeted therapy—can improve clinical prognosis [11,44,61]. Unfortunately, it is not uncommon for a small number of patients to be diagnosed with high grade stage III BC (where tumours are found in axillary or sentinel lymph nodes) at the time of initial diagnosis [3,62]. BC has four main stages and is dependent on the size, type and invasion of the tumour, where stage 0 is non-invasive and stage IV is an advanced metastatic disease that has spread to other organs [3]. Current imaging techniques (summarised in Table 1) to identify cancer may lack the appropriate balance of sensitivity and specificity in detecting lesions, which can lead to underdiagnosis or misdiagnosis [44,61,63,64].

Although current medical imaging modalities are capable of detecting tumour masses, the lack of sensitivity and specificity proves it is difficult to diagnose early stage breast tumour malignancies [65]. Contrast agents (CAs) can enhance the imaging capabilities of medical imaging modalities to differentiate between benign and malignant tissues [66,67]. CAs act as imaging probes that bind to the tissues/cells of interest and present them with greater conspicuousness on images, by altering how the signal or energy is exhibited by the pathology compared to surrounding healthy tissue [68,69]. While CAs can be used to improve image quality by enhancing the image contrast between pathological and normal tissues, they are not tumour-specific or pathology-specific and can exhibit non-specific biodistribution [70,71,72,73,74,75,76]. In addition, conventional imaging techniques are only capable of detecting abnormal breast lesions, and thus are unable to provide further information and characteristics of the molecular profiles of BC which could improve clinical outcomes [13]. Consequently, targeted imaging using molecular expression patterns of tumours, such as HER2, is urgently required to better detect and diagnose this aggressive BC.

## 4. Molecular Imaging

The principle of molecular imaging is based on the visualisation and measurement of biological and physiological processes that occur at the cellular level using exogenous CAs, which enables sensitive detection of tumours [65]. Molecular imaging enables clinicians to not only detect the locations of tumours within in the body, but also assess the expression profiles and biological processes that influence tumour development and responses to therapy [65]. Relevant imaging modalities commonly used for molecular imaging include positron emission tomography (PET) and single photon emission computed tomography (SPECT) [65,77]. Both PET and SPECT operate through the detection of γ-rays from radiolabelled agents. PET uses positron emitting radioisotopes: fluorine-18 (^18^F), oxygen-15 (^15^O), nitrogen-13 (^13^N) or carbon-11 (^11^C). SPECT utilises single photon emitting radioisotopes: technetium-99m (^99m^Tc), iodine-123 (^123^I) or iodine-125 (^125^I) [65]. These radioisotopes act as signalling agents when bound to a targeting moiety, such as trastuzumab, a known HER2 antibody [68,78]. Furthermore, PET can also be integrated with CT (PET/CT) or with MRI (PET/MRI) to further increase sensitivity and specificity when detecting primary tumours and lymph node metastases [64,79].

One of the earliest positron-emitting radioisotopes used for PET imaging was gallium-68 [80]. However, early ^68^Ga generators produced [^68^Ga]Ga-ethylenediaminetetraacetic acid (EDTA) conjugates, which would further require destruction of the complex in order to prepare the radiopharmaceuticals, leading to time-consuming processes and overall poor yields [80]. As a result, gallium-68 used in PET was sidelined, and other radiopharmaceuticals containing fluorine-18 and technetium-99 became popular. More recently, there has been an up-surge in PET imaging with gallium 68 due to the convenience of on-site synthesis and elution of the radioisotope from a germanium-68/gallium-68 generator [81,82]. The compact design of germanium-68/gallium-68 generators mean they can be housed in most nuclear medicine imaging laboratories without requiring modifications to support their use, and also enables gallium-68 production on-demand [80,81]. Furthermore, the ease and compact size of a germanium-68/gallium-68 generator add to its cost-effectiveness and efficient production when compared to other PET radioisotopes, such as fluorine-18, nitrogen-13, carbon-11 and oxygen-15, which are cyclotron produced [82,83].

One such molecule that is radiolabelled to enhance imaging specificity and sensitivity in detecting tumours is the ligand Glu-NH-CO-NH-Lys-(Ahx)-(HBED-CC), which binds to prostate-specific membrane antigen (PSMA) [84,85]. PSMA is a transmembrane protein that is overexpressed one-hundred to one-thousand times higher in malignant prostatic tumours compared to healthy prostate tissue [81,84]. In a retrospective analysis of PSMA scans by Kallur et al. [84], where HBED-CC was radiolabelled with gallium-68, a γ-emitting radioisotope; [^68^Ga]Ga-HBED-CC-PSMA demonstrated 95% sensitivity in baseline diagnosis and 98% positive predictive value. Furthermore, [^68^Ga]Ga-HBED-CC-PSMA was able to detect disease progression in 100% of patients who had surgical castration [84,85]. In a separate randomised study, Hofman, et al. [86] compared the sensitivity and specificity of [^68^Ga]Ga-HBED-CC-PSMA PET/CT to those of conventional CT imaging and bone scans in the detection of metastatic disease. Although participants were randomised for imaging, they were later crossed-over unless three or more distant metastases were identified [86]. This study demonstrated that [^68^Ga]Ga-HBED-CC-PSMA PET/CT was more specific and sensitive than combined CT and bone scans in the detection of metastatic disease, with [^68^Ga]Ga-HBED-CC-PSMA PET/CT yielding 27% greater accuracy (92% vs. 65% for conventional imaging) [85,86]. Thus, it was concluded that [^68^Ga]Ga-HBED-CC-PSMA PET/CT is suitable for replacing conventional imaging techniques for the detection and staging of prostate cancer patients.

While the previous studies demonstrate the versatility of molecular imaging, Shetty et al. [87] highlighted the potential non-specific uptake of [^68^Ga]Ga-HBED-CC-PSMA in non-prostatic tissues: renal cell cancers, reactive lymph nodes, the gall bladder, accessory salivary glands, celiac ganglia, non-small cell lung cancers and neuroendocrine tumours. This is due to PSMA also being expressed on tissues such as kidney, small intestine and salivary gland tissues, which can take up [^68^Ga]Ga-HBED-CC-PSMA, leading to potential false upstaging or downstaging of prostate cancer, indicating the lack of specificity for the disease [87]. As a result, agents with greater specificity to the disease are required to improve the accuracy of cancer detection and diagnosis.

### 4.1. HER2-Targeted Molecular Imaging with Monoclonal Antibodies

**Figure 1:**
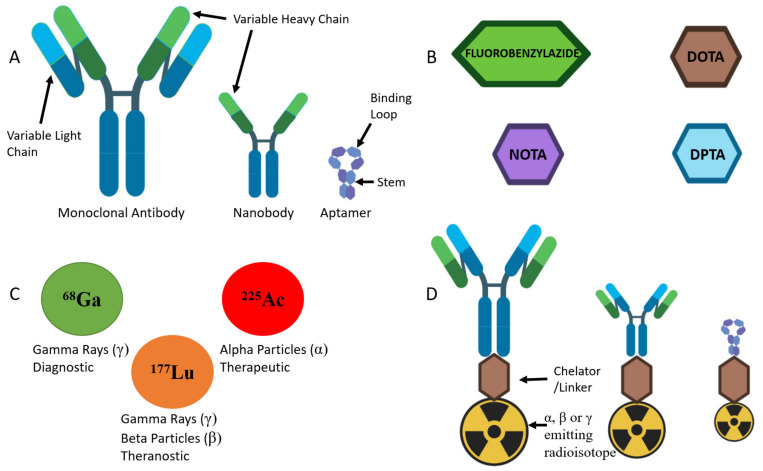
A diagram of targeting ligands, chelators, radionuclides and conjugated radiopharmaceuticals. (**A**) A schematic representation of monoclonal antibody, nanobody and aptamer structures. Antibodies each consist of two identical heavy chains and two identical light chains which are involved in recognising a cognate target. Nanobodies each consist of only two identical heavy chains and lack light chains. Aptamers each contain a binding loop which recognises and binds to the target. (**B**) Commonly used chelators and linkers attach radionuclides to targeting ligands. (**C**) Different types of radioisotopes are used for diagnostic, therapeutic and theranostic applications. (**D**) Radioisotopes are attached to targeting ligands (antibodies, nanobodies, aptamers) with linker molecules, which can then be used for diagnostic, therapeutic or theranostic functions. Adapted from [22,93,94].

HER2-targeted molecular imaging was investigated in preclinical and clinical studies with radiolabelled monoclonal antibodies (mAbs) and nanobodies [13]. In molecular imaging, mAbs are conjugated to radioisotopes (Figure 1), using a chelator as a linker, to function as imaging probes [68]. Common chelators that are used to link mAbs and radioisotopes together include 1,4,7,10-tetraazacyclododecane-1,4,7,10-tetraacetic acid (DOTA), 1,4,7-triazacyclononane-1,4,7-triacetic acid (NOTA) and diethylene triamine pentaacetic acid (DPTA) [68,88]. Radiolabelled mAbs are currently being investigated due to their ability to target molecular biomarkers with high specificity and sensitivity [68,89,90,91]. Results obtained from completed clinical trials evaluating the efficacy of positron-emitting radioisotopes conjugated to antibodies for PET imaging have demonstrated high sensitivity and specificity in detecting cancer [92].

A study by Laforest et al. [78] evaluated the efficacy of a zirconium-89 (^89^Zr) radiolabelled HER2-antibody, trastuzumab, in patients with HER2-positive BC, and concluded that [^89^Zr]Zr-trastuzumab produced optimal PET/CT images with the best available tumour-to-non-tumour contrast, four days post-injection. Increased [^89^Zr]Zr-trastuzumab uptake was observed in a minimum of one known lesion in 10 out of 12 patients and at least one lesion in six patients with metastatic disease [78]. Interestingly, Ulaner et al. [93] investigated the potential of [^89^Zr]Zr-trastuzumab to detect HER2(+) metastases in patients with HER2(-) primary BC. Biopsy-confirmed HER2(-) BC patients (*n* = 9) were screened with [^89^Zr]Zr-trastuzumab PET/CT for HER2(+) distant metastases[95]. In five patients, suggestive foci on [^89^Zr]Zr-trastuzumab PET/CT were observed; however, a biopsy showed no evidence of HER2(+) disease in three of those participants. This false positive may have occurred due to the non-specific uptake of free ^89^Zr, which preferentially accumulates in bones, in osseous lesions, suggesting the low specificity of [^89^Zr]Zr-trastuzumab under certain conditions [95]. Interestingly, in the remaining two patients with suggestive foci on [^89^Zr]Zr-trastuzumab PET/CT, biopsies confirmed the presence of HER2-positive metastasis, indicating the potential of radiolabelled HER2 antibody to detect unsuspected HER2 distant metastasis in HER2-negative primary BC [95]. However, not only are radiotracers with greater specificity required to reduce false-positive rates, but the selection of an appropriate radioisotope with fast target localisation and rapid blood clearance to improve tumour-to-background ratio is also needed [80,95].

Challenges remain, however, with the conjugation of antibodies to the imaging agent, as the modification process to conjugate an imaging agent to the antibodies can compromise its specificity, functionality and affinity for to the target epitope [68]. Furthermore, resistance to trastuzumab poses an obstacle to targeted therapy, as patients may not respond to the drug [91,96]. Primary resistance to trastuzumab occurs in a significant number of patients (up to 35%), due to an inactive target, which could potentially result in false negative tumour detection [91,97,98]. Similarly, in cases of acquired resistance, where patients develop resistance to trastuzumab due to prolonged therapy, false negatives could occur, as trastuzumab may not be able to bind to its target [96,98]. Acquired resistance may be a result of steric hindrance of receptor-antibody interactions by other receptor proteins, or mutated isoforms of truncated HER2 receptors which lack the extra-cellular domain (ECD) binding epitope for recognition by trastuzumab [96,98].

### 4.2. HER2-Targeted Molecular Imaging with Nanobodies

While antibodies have demonstrated potential in oncology and nuclear medical imaging, the inherent large size of mAbs (four polypeptide chains, 150 kDa) can limit their access to tumour cells [99]. Antibodies of camelids (camels, alpacas, llamas) are devoid of light chains, which contributes to their reduced size and their name, nanobodies [99]. Additionally, nanobodies have lower immunogenic profiles due to similar homology (>80%) in sequence identity to the human Ig3 family [93]. Given the desirable properties of nanobodies, they are more advantageous than mAbs for oncology applications delivering therapeutics to tumour sites [93,94]. In addition, the short half-lives of nanobodies in blood circulation enable unbound nanobodies to be removed quickly from the body after administration, assuring a high tumour-to-background ratio [93,100]. Similarly to mAbs, nanobodies can be radiolabelled with a radioisotope, fluorescent dye or microbubble to allow imaging technologies to sensitively and specifically detect and locate cancer cells [101].

A phase I study of an anti-HER2-nanobody radiolabelled with gallium-68 (^68^Ga) was investigated for PET/CT assessment of HER2 expression in patients with primary and metastatic BC [102]. In total, 20 patients were administered [^68^Ga]Ga-anti-HER2-nanobodies and assessed with PET/CT scans at 10, 60 and 90 min intervals. Uptake of radiotracer was observed in primary lesions, along with rapid blood clearance—only 10% of injected activity remaining one hour after administration [102]. Although accumulation of [^68^Ga]Ga-anti-HER2-nanobody was observed in kidneys, liver and intestines, it was observed that in patients with HER2 metastases, uptake of the radiotracer was high compared to normal surrounding tissues and radioactivity was well above background levels [102]. In the absence of adverse effects, Keyaerts et al. [102] concluded that radiolabelled anti-HER2-nanobodies are safe for use in humans and deserve phase II clinical trials. However, their rapid clearance rates may limit the use of nanobodies as imaging probes, as they may be rapidly cleared before reaching their cognate targets [93]. As a result of fast renal clearance rates and high accumulation in kidneys, their use for detecting lesions in organs near the kidney, such as the pancreas, may be limited [93,101].

Although nanobodies derived from camelids share only >80% homology with the V_H_ chains in human antibodies, there is the potential for the foreign proteins to illicit an immune response from patients [93,101,103]. The immunogenicity response could be minimised through “humanisation”—replacing surface regions or variable regions (in mAbs) with human sequences [94]. However, this modification may compromise functionality, negate the intended immunogenicity-lowering effects of the nanobodies due to decreased solubility or induce human anti-human antibody responses to the humanised mAbs [94,104,105]. Given those challenges and drawbacks of mAbs and nanobodies, there is a clear need for a novel agent with less limitations. Novel agents such as aptamers can overcome the drawbacks of mAbs and nanobodies to improve BC diagnosis in molecular imaging [24,25,28].

## 5. Aptamers

Aptamers are oligomers consisting of single-stranded RNA or DNA nucleotides, 20–100 base pairs in length, which are capable of binding to selected targets with remarkable specificity and affinity [18,19,20,21]. Due to their similarities in specifically recognising and binding to their targets via an induced-fit mechanism, aptamers are known as ‘chemical antibodies’ to distinguish them from their proteinaceous counterparts [24,25,28]. Aptamers have the ability to form unique secondary and tertiary structures through intra-nucleotide bindings: hydrogen bonds, hydrophobic interactions and van der Waals interactions, which enables them to specifically recognise and bind to their complimentary targets with great affinity [24,28]. Compared to their larger protein antibody counterparts, which are constrained by potential toxicity and immunogenicity, aptamers are not limited by their physical and chemical characteristics in clinical applications, which include diagnostics, therapeutics and theranostics [20,24,27,28]. Due to their smaller size (10 to 20 kDa), aptamers have greater tumour penetration potential for improved treatment efficacy regarding solid tumours, and may also bind to hidden epitopes that are restricted to larger antibodies (~150 kDa) [25,106]. Furthermore, aptamers are sequence defined and chemically synthesised, which enables cost-effective, highly consistent large-scale production and reproducible synthesis [20,24].

Aptamers are generated by an in vitro selection process known as ”systematic evolution of ligands by exponential enrichment (SELEX)”, where a large pool of randomised nucleic acid sequences are iteratively incubated with the target molecule [18,19,21]. During the processes of binding, selection, separation and amplification, the enrichment of high binding affinity oligonucleotides is enhanced by specifically selecting the bound sequences to be amplified in the subsequent selection cycles [24,28]. As a result, aptamers can be generated to recognise a wide range of molecular targets and be selected against disease-related biomarkers for diagnostics and therapeutic applications [20,107,108,109,110]. Previous research has indicated the potential of numerous aptamers capable of specifically targeting various types of cancers—leukaemia, glioblastoma, lung cancer and breast cancer. They could be utilised in clinical settings for molecular imaging (diagnostics) and targeted therapeutic applications [25,27,28,46,107,108,110,111,112,113,114,115,116].

### 5.1. Aptamers in Targeted Theranostics

While conventional anticancer therapies are effective at killing cancer cells, their lack of tissue selectivity and specificity can also affect healthy cells, resulting in unwanted toxicity and side effects [61,63,117]. The greatest advantage demonstrated by aptamers, compared to antibodies, is their ability to still retain high target specificity and affinity after being functionalised [20,25,106,110]. The ease of chemically modifying aptamers to carry and deliver therapeutic payloads (cytotoxic drugs, siRNA and radioisotopes), coupled with the ability to generate them against a wide-range of cancer biomarkers, makes them a promising tool for oncology treatment, molecular imaging and diagnostics [20,22,25,107,114,118,119,120]. After binding to targeted surface receptors, aptamers are internalised with their payloads to exert their effects [27,46,121]. Previous research with radiolabelled aptamers (summarised in Table 2) has demonstrated efficacy in recognising targets for molecular imaging, when conjugated with specific radioisotopes such as copper-64 (^64^Cu), ^18^F and ^68^Ga [22,118,122,123,124]. Thus, aptamers can be radiolabelled and functionalised with different radioisotopes using chelators or by applying strategies of radiolabelling antibodies and nanobodies to aptamers [22,118]. The functionalisation of aptamers with radiolabels via different chelators does not appear to compromise their target affinity, which makes them an attractive agent in oncology [22,118]. While the addition of a chelator to an aptamer does not appear to affect its specificity for its target, the radiolabelling efficiency and yield may be dependent on the type of chelator (under the same conditions) [122].

#### 5.1.1. Diagnostic Applications

As for the diagnostic applications of aptamers, nuclear medicine imaging techniques with radiolabelled aptamers can achieve highly sensitive and specific real-time molecular imaging for cancer detection [23,118]. Although there are numerous radioisotopes available for nuclear imaging, each radioisotope has a characteristic half-life, decay mode and production method, and characteristic chemical properties [118]. Furthermore, the modes of nuclear medicine imaging techniques also vary in sensitivity, with PET being ten-fold more sensitive than SPECT for imaging molecular processes [119]. Therefore, it is important to consider both the characteristics of both the radioisotopes and aptamers, circulation half-life and in vivo stability, to target diseases for early detection and diagnosis [129].

Given the advantages of PET imaging with ^68^Ga, this radioisotope is a desirable choice with nuclear medicine imaging techniques for early cancer detection. Gallium-68 radiolabelled molecules in PET/CT has been improving nuclear medicine imaging in terms of the detection of metastatic disease [81,84,130].

Successful radiolabelling of aptamers has been demonstrated to not affect aptamer target binding affinity [118,122]. Rockey et al. [122] investigated the parameters for optimal radiolabelling RNA aptamers with different chelators: DOTA-NHS, NOTA-NHS, 3,6,9,15-tetraazabicyclo [9.3.1]pentadeca-1(15),11,13-triene-3,6,9-triacetic acid (PCTA) and 3,6,10,13,16,19-hexaazabicyclo[6.6.6] icosane-1,8-diamine (diAmSar), to develop [^64^Cu]Cu-labelled aptamers for PET imaging. After radiolabelling the RNA aptamers which are specific for PSMA, the [^64^Cu]Cu-NOTA and [^64^Cu]Cu-PCTA RNA aptamers demonstrated significantly greater binding affinity to the PSMA-positive prostate cancer cells, 22Rv1[1.7], than the PSMA-negative prostate cancer cells, PC-3, similarly to the unconjugated radiolabelled RNA aptamer [122]. These results suggest that the radiolabelling and the chelators did not alter the specificity of the aptamers. Similarly, Li et al. [125] characterised the cellular uptake of a [^64^Cu]Cu-labelled AS1411 nucleolin aptamer using four different chelators, DOTA, S-2-(4-Isothiocyanatobenzyl)-DOTA (DOTA-Bn), Cross-bridged 4,11-bis(carboxymethyl)-1,4,8,11-tetraazabicyclo[6.6.2]hexadecane (CB-TE2A) and NOTA-Bn, in H460 human non-small cell lung tumour cells. In vitro analysis demonstrated that all conjugates, [^64^Cu]Cu-DOTA-AS1411, [^64^Cu]Cu-CB-TE2A-AS1411, [^64^Cu]Cu-DOTA-Bn-AS1411 and [^64^Cu]Cu-NOTA-Bn-AS1411, were taken up by the H460 cells [125]. The amount of radiolabelled AS1411 taken up by chelators decreased in the following order: DOTA-NHS > CB-TE2A > DOTA-Bn > NOTA-Bn, suggesting that a suitable chelator is required for reasonable uptake of radiolabelled aptamers [125]. Interestingly, in vivo biodistribution studies demonstrated PET/CT imaging with [^64^Cu]Cu-CB-TE2A-AS1411 resulted in tumours being visible from 1 h to 24 h post-injection, unlike the lack of tumour visibility by [^64^Cu]Cu-DOTA-AS1411 at 24 h post-injection. Furthermore, [^64^Cu]Cu-CB-TE2A-AS1411 exhibited an 80% faster clearance rate at 1 h post-injection while maintaining a greater tumour-to-muscle ratio within 24 h compared to [^64^Cu]Cu-DOTA-AS1411 [125]. The uptake of the radiolabelled aptamers with different chelators indicates that the specificity of the aptamers remains uncompromised; however, the percentage uptake is dependent on the type of chelator attached.

Jacobson et al. [131] investigated the PET imaging capabilities of ^18^F and ^64^Cu radiolabelled tenascin-C aptamers. Both [^18^F]F- N-succinimidyl 4-18F-fluorobenzoate (FSB)-tenascin-C and [^64^Cu]Cu-NOTA-tenascin-C aptamers demonstrated significantly greater uptake in tenascin-C-positive tumours, U87MG and MDA-MB-231, compared to the tenascin-C-negative tumours, H460, suggesting that the aptamers did not lose their target specificity when radiolabelled with copper-64 for PET imaging in vivo [131] Thus, [^68^Ga]Ga-aptamers could provide highly specific and sensitive cancer detection and therefore improve patient outcomes with advantages of speed and cost-efficiency [118]. While they are beyond the scope of this review article, other studies have investigated radiolabelling various aptamers to target cancer biomarkers for molecular imaging [128,132,133,134,135,136,137,138]. Further, mAb radiolabelling techniques can be transferred to aptamers with ease to functionalise them as molecular imaging agents [22].

#### 5.1.2. Therapeutic Applications

The ease of functionalising aptamers by conjugation with a versatile range of functional groups enables them to serve as targeted delivery vehicles for therapeutic treatments of cancer [139,140]. Aptamers conjugated with chemotherapeutics or therapeutic radioisotopes enable targeted therapeutic treatment for cancers, while limiting off-target toxic effects to healthy tissues and consequent adverse side effects [139]. Thus, aptamers have been extensively researched and studied as targeted therapeutics that deliver cytotoxic drugs or high-energy radioisotopes [139].

As drug delivery vehicles, a HER3 aptamer selected via SELEX for the HER3 extracellular domain (ECD) by Dou et al. [141] was investigated for targeted delivery of doxorubicin (DOX), a broad-spectrum chemotherapeutic, to HER3(+) MCF-7 and BT474 BC cell lines. The aptamer was functionalised with liposome encapsulating DOX (Apt-lip-DOX), and its efficacy was investigated against liposome-DOX (lip-DOX) and free-DOX. The Apt-lip-DOX conjugate was demonstrated to have better inhibited the growth of MCF-7 and BT474 cells, when compared to the lip-DOX and free-DOX treatments. In vivo studies of the Apt-lip-DOX conjugate demonstrated significantly greater uptake and prolonged retention in MCF-7-tumour-bearing mice when compared to lip-DOX and free-DOX groups [141]. Furthermore, mice that were treated with Apt-lip-DOX conjugates had significantly smaller tumours when compared to the control (NaCl treatment), lip-DOX and free-DOX groups, suggesting the uptake of DOX was further enhanced and facilitated by the aptamer targeting the overexpression of HER3 on the cell surfaces of the tumours [141].

Similarly, Macdonald et al. [121] investigated a bifunctional aptamer (TEPP) targeting the transferrin receptor on the blood–brain barrier (BBB) and the epithelial adhesion molecule (EpCAM) on metastatic cancer cells, to selectively deliver DOX to EpCAM positive tumours. Mice were inoculated with a brain-metastatic variant of the MDA-MB-231 (MDA-MB-231Br) BC cell line with known EpCAM expression, and when brain metastasis was detected, the mice were treated with TEPP, TENN (negative control aptamer), TEPP-DOX or TENN-DOX. Although an aptamer signal was found in all treatment groups, the colocalization signal from the TENN aptamers was limited, whereas the TEPP aptamers generated clear fluorescent signals on the GFP (green fluorescent protein)-positive tumour cells [121].

Bandekar et al. [142] investigated the targeted delivery of radiopharmaceuticals using radiolabelled anti-PSMA antibodies and A10 PSMA aptamers for targeted radiotherapy. Banekar et al. [142] evaluated the efficacy of anti-PSMA antibody J591 and A10 PSMA aptamer conjugated with α-particle-generating, actinium-225-loaded liposomes in PSMA(+) LNCaP and Mat-Lu cells. The results of this study demonstrated that [^225^Ac]Ac-lip-A10 exhibited significantly greater cytotoxicity compared to non-targeted [^225^Ac]Ac-lip in PSMA(+) prostate cancer cells, suggesting the specific delivery of the radioisotope [142]. However, it is worth noting that the [^225^Ac]Ac-lip-J591 antibody exhibited the greatest cytotoxicity, compared to [^225^Ac]Ac-lip-A10 aptamer and [^225^Ac]Ac-lip. However, these results should be taken cautiously, as a characterisation of liposome loading and conjugation revealed that there were approximately 17 ± 2 J591 antibodies per liposome compared to the 9 ± 2 A10 aptamers per liposome in 2.5 μmol of total liquid [142]. Furthermore, typical aptamer-drugs conjugates usually comprise three parts: aptamer ligands, linker molecules and drug moieties, as opposed to being loaded into liposomes [139,142]. Since the A10 PSMA aptamer was radiolabelled with actinium-225 and demonstrated promising results as a targeted drug delivery system, this study provides proof-of-concept evidence for HER2 aptamers to be radiolabelled and produce a therapeutic effect.

Functionalising aptamers with various functional groups such as cytotoxic drugs or high energy radioisotopes would enable them to become highly efficacious targeted delivery vehicles for cancer treatments. Furthermore, by providing highly targeted therapy, the risk of off-target effects could be lowered, and the side effects experienced when undergoing cancer therapy could potentially be reduced [139]. As a result, it is worth further investigating highly specific targeting ligands, such as aptamers, conjugated with therapeutic radioisotopes to improve cancer treatment and prognosis.

## 6. HER2 Aptamers

Regarding molecular imaging probes and targeted therapy, radiolabelled aptamers are promising radiopharmaceuticals for nuclear medical imaging [118,143]. Several HER2 DNA/RNA aptamers have previously been developed and reported to target HER2 receptor in HER2+ BC with great specificity and selectivity, as summarised in Table 3 [8,9,27,28,46,116].

The HER2 DNA aptamer, HB5, was developed by Liu et al. [46] via SELEX against purified HER2-peptides immobilised to carboxylated magnetic beads using an FITC-labelled ssDNA library. The initial ssDNA pool contained sequences of 86 nucleotides; each sequence consisted of 40 random nucleotides flanked by two constant sequences for PCR amplification reaction [46]. In the study, the HB5 aptamer demonstrated high binding capacity to the HER2 peptide and the extracellular domain of the HER2 protein. The HB5 aptamers demonstrated specific binding to the HER2-positive SK-BR-3 cell line, and non-specific, weak binding to the TNBC MDA-MB-231 cell line, when analysed by flow cytometry. However, it is unclear what the binding affinity was to each cell line. The HB5 was also investigated as a tumour-targeted delivery vehicle to carry DOX to HER2 + BC cells. While uptake of free DOX was demonstrated by both cell lines, SK-BR-3 and MDA-MB-231, selective uptake of the HB5-DOX conjugate was mainly observed in HER2 + cells, and it had reduced cytotoxicity in HER2(−) cells [46]. In contrast to the HER2 aptamers identified (Table 3), the HB5 aptamer was only investigated for its targeted drug delivery system and was not radiolabelled to investigate its nuclear medical imaging specificity for HER2 BC detection and diagnosis [46].

## 7. HER2 Aptamers and Nuclear Medicine Imaging

Gijs et al. [124] developed two DNA aptamers to target the HER2 receptor using a whole-cell SELEX approach. The two aptamers, HeA2_1 and HeA2_3, demonstrated specific binding to HER2(+) cell lines (SKOV3 and SK-BR-3) and HER2(+) tumours when compared to the MDA-MB-231 cell line. HeA2_3 demonstrated internalisation by SKOV3 (ovarian adenocarcinoma) by comparing the cellular localisation of the aptamer against that of the anti-HER2 antibody [124]. Internalisation of the aptamer was confirmed by local fluorescent intensity in the cytoplasm, unlike the anti-HER2 antibody localised at the cell-surface (characteristic localisation of HER2 receptors) [124]. Interestingly, HeA2_1 and HeA2_3 also inhibited cell-proliferation of SKOV3 cells by 1.26-fold and 1.30-fold respectively, when compared to untreated cells and the negative control aptamer [124]. Treatments of HeA2_1 and HeA2_3 on HER2(-) cell line did not demonstrate significant inhibitory effects on cell number, thereby suggesting that the mechanism of action may be related to the HER2 receptor.

To further investigate the potential of HER2 aptamers for molecular imaging in vivo, Gijs et al. [124] modified the aptamers into PET imaging agents by radiolabelling HeA2_1 and HeA2_3 with gallium-68 via a NOTA-chelator and evaluating the ex vivo biodistribution and tumour-targeting properties. Interestingly, [^68^Ga]Ga-labelled HeA2_1, HeA2_3 and negative control aptamer uptake were 1.5-fold higher in SKOV3 tumours than MDA-MB-231 cells, and no significant differences were observed between the HER2 aptamers and the negative control, suggesting tumour uptake was due to tumour factors other than HER2 expression [124]. Rapid accumulation and release of the radiolabelled aptamers was detected in the liver, bladder and kidneys, reflecting renal clearance. In contrast to the decrease in radioactivity in healthy tissues, accumulation of radiolabelled aptamers in HER2(+) tumours, as measured by standard uptake values (SUV), continued to increase over selected time points over approximately 60 min [124]. This observation of [^68^Ga]Ga-NOTA-aptamers is in contrast to the [^68^Ga]Ga-NOTA-HER2-nanobody, for which maximum uptake was observed after 10 min and decreased over time [102]. As a result of no significant differences between the uptake of the radiolabelled HeA2_1 and HeA2_3 and the negative control aptamer, this suggests that accumulation and uptake by tumours may be results of binding affinity and other factors, as opposed to specificity to HER2 [124]. Additionally, the greater tumour penetrance may have been due to the smaller size of aptamers [144]. Furthermore, it should be noted that high levels of radioactivity were present in blood protein, which suggests non-specific interactions may occur in vivo [124].

Kim et al. [27] evaluated a commercial HER2 aptamer, SH-1194-35, developed by Aptamer Sciences Inc. (from South Korea) for its potential applications in nuclear medical imaging. Its binding characteristics and specificity for HER2 were verified by in vitro flow cytometry and confocal microscopy. SH-1194-35 demonstrated high binding to the HER2(+) BT474 cell line, and non-specific, low binding to MDA-MB-231 [27]. The level of HER2 expression in each cell line was confirmed using a positive control, HER2(+) SK-BR-3, and a negative control, HER2(−) HS578T cell line. Fluorine-18 was conjugated via a FSB linker, to the aptamer ([^18^F]F-FSB-SH-1194-35) to determine its application for in vivo PET imaging. Whole body PET imaging of tumour-bearing mice demonstrated greater tumour uptake of [^18^F]F-FSB-SH-1194-35 in BT474 cancer cells, compared to MDA-MB-231 tumours, indicating the specificity of the radiolabelled aptamer for targeting HER2 in vivo [27]. Interestingly, ex vivo examination and PET imaging both showed high levels of [^18^F]F-SFB-SH-1194-35 accumulation in the bowel and bladder region, despite significantly higher uptake in HER2(+) tumours, reflecting the rapid excretion pathways of the aptamers [27].

Zhu et al. [123] developed several HER2 aptamers using a combined protein-based SELEX and whole cell-SELEX. The ssDNA library was initially screened against the HER2-ECD and the generated pool of DNA sequences was then subsequently screened against live HER2(+) SKOV3 cells; and the seven most frequent sequences, termed Heraptamer1 to Heraptamer7, were selected for in vitro characterisation and in vivo screening [123]. All seven Heraptamers not only demonstrated binding to HER2-coupled beads, but had enhanced fluorescent intensity when incubated with SKOV3 cells, indicating strong binding affinity to HER2(+) tumours [123]. The specificity of Heraptamers 1–7 was tested against three different HER2(−) cell lines, MDA-MB-231, MDA-MB-435 and MCF7, and they were found to not bind to any of those cell lines, demonstrating their specific binding to HER2 [123]. The aptamers Heraptamer1 and Heraptamer2 were selected for PET imaging of HER2 cancer mouse models, after in vivo screening using SKOV3 xenograft tumour demonstrated high tumour uptake when radiolabelled with ^18^F[F]. While biodistribution analysis showed high levels of accumulation in the kidneys and liver, reflecting the excretory pathways, significant tumour uptake of both ^18^F[F] radiolabelled Herpaptamer1 and Heraptamer2 was also observed with high signal-to-background ratios, indicating promising imaging potential. Interestingly, Zhu et al. [123] also demonstrated the specificity of the [^18^F]F-fluorobenzyl-azide-heraptamer1 and [^18^F]F-fluorobenzyl-azide-heraptamer2, by blocking them with corresponding unlabelled aptamers which significantly reduced ^18^F[F] radiolabelled aptamers in SKOV3 tumours. Further, the specificities of the [^18^F]F-fluorobenzyl-azide-Heraptamer1 and [^18^F]F-fluorobenzyl-azide-Heraptamer2 were demonstrated by the low tumour uptake ratios in HER2-negative MDA-MB-231 tumour [123]. It is noteworthy that Zhu et al. [123] did not test the Heraptamers on HER2(+) breast cancer cell lines such as SK-BR-3, as the previously mentioned studies did. However, the results obtained from the combined HER2-ECD screening and HER2(+) cells indicate that Heraptamer1 and Heraptamer2 are potential ligands for HER2 imaging in cancer.

Therefore, aptamers can be functionalised with different radioisotopes for specific clinical applications in nuclear medicine [118]. For example, conjugation with gallium-68 or fluorine-18 can provide diagnostic applications due to the emission of γ-rays [118]. Targeted radioisotope therapy to eradicate tumour cells while sparring neighbouring healthy tissue with actinium-225 due to the short range of high-dose ionising α particles emitted can be utilised [145]. Combined therapeutic and diagnostic (theranostic) applications with lutetium-177 conjugation due to its simultaneous emission of γ-rays and β-particles can be used for detection and treatment respectively [118,146].

## 8. Potential Therapeutic Applications of HER2 Aptamers

Alpha-particle emitting radioisotopes are emerging as promising and effective therapeutic radioisotopes for targeted treatment of many types of cancers [147,148]. They offer key advantages over β-particle radiation and conventional radiotherapy, due to high linear energy transfers (LET) and short path lengths in human tissue [149,150]. Alpha-particles can produce more lethal double-stranded DNA breaks and DNA cluster breaks per volume than β-particles (a single alpha-particle can kill a cell, compared to thousands of beta-particles required for similar effect) due to the high LET of α-particles (α = 100 keV/µm versus β = 0.2 keV/µm) [148,149,150]. The short range of α-particles corresponds to only a few cell diameters (47 to 85 µm), which enables them to selectively kill targeted cancer cells while limiting off-target damage and sparing healthy tissues [148,149]. This is advantageous to both conventional radiotherapy, which requires an external radiation beam delivered to the tumour site which also exposes healthy tissue to ionising radiation, and the relatively longer path range (0.5–12 mm) in tissue of β-radiation, which also deposits energy into both the cancer cells and surrounding healthy tissue [148,150,151].

While there are many α-particle-emitting radioisotopes, only a few meet the criteria for therapeutic use and have been investigated in pre-clinical and clinical studies [148]. Actinium-225 (^225^Ac) is an α-particle emitter that can be obtained from the decay of uranium-233 (^233^U) [150]. Actinium-225 has a relatively long half-life of 9.9 days; however, its decay cascade to stable bismuth-209 (^209^Bi) yields six daughter radioisotopes [149,150]. In total, the main actinium-225 decay cascade path yields net four α-particles (5.8 to 8.4 MeV) and two beta disintegrations of high energy (0.6 and 1.6 MeV), with co-emissions of γ-rays from francium-221 (^221^Fr) and bismuth-213 (^213^Bi) daughter radioisotopes that can also be used for imaging purposes [149].

In a clinical study by Sathekge, et al. [152], the therapeutic efficacy of [^225^Ac]Ac-PSMA-617 was assessed in chemotherapy-resistance patients with advanced stage prostate cancer. Patients with metastatic lesions and sufficient PSMA expression, as determined by [^68^Ga]Ga-PSMA-11 PET/CT, were selected for [^225^Ac]Ac-PSMA-617 treatment. [^225^Ac]Ac-PSMA-617 demonstrated significant therapeutic efficacy in chemotherapy-resistance patients, with 82% of patients exhibiting >90% declines in PSA, and 41% having undetectable serum PSA levels [152]. Although mild to moderate xerostomia was experienced by patients, no acute toxicity was observed, indicating that [^225^Ac]Ac-PSMA-617 is relatively safe and warrants further investigations into actinium-225 radiation therapy [152]. To determine the therapeutic efficacy of [^225^Ac]Ac-labelled HER2 nanobody, 2Rs15d, Pruszynski et al. [153] evaluated the binding specificity, affinity, internalisation, physiologic stability and toxicity in HER2(+) cells in vitro. The radiolabelled nanobody, [^225^Ac]Ac-DOTA-2Rs15d, demonstrated high binding affinity and specificity for HER2(+) SKOV-3 cells, and negligible binding to HER2(−)MDA-MB-231 cells [153]. Additionally, with increasing concentrations and incubation times, [^225^Ac]Ac-DOTA-2Rs15d demonstrated significant reductions in cell-viability when compared to [^225^Ac]Ac-DOTA in SKOV-3 cells [153]. Contrastingly, non-significant differences in cell viability between [^225^Ac]Ac-DOTA-2Rs15d and [^225^Ac]Ac-DOTA treatments were observed in MDA-MB-231, indicating that cytotoxicity was directly correlated with HER2 expression levels [153]. Biodistribution analysis conducted by Pruszynski et al. [153] also found the uptake of [^225^Ac]Ac-DOTA-2Rs15d in SKOV-3 tumour bearing mice to be 8-fold greater than that of MDA-MB-231 after 2 h, and further increased to 15-fold after 48 h, indicating the specificity of [^225^Ac]Ac-DOTA-2Rs15d for HER2.

As previously discussed, Bandekar et al. [142] demonstrated the ability of aptamers to be radiolabelled with therapeutic radionuclides, which suggests the potential for HER2 aptamers to be functionalised in a similar fashion. As a result, radiolabelling HER2 aptamers with actinium-225 to produce [^225^Ac]Ac-aptamers could provide highly targeted and efficacious cancer treatments and improve patient prognosis in not only people suffering from primary and metastatic cancers, but also chemotherapeutic and conventional radiotherapy-resistant patients.

## 9. Potential for Theranostic Applications

Theranostic applications with lutetium-177 (^177^Lu) are gaining attention in targeted radioisotope therapy due to its unique, simultaneous emissions of γ-rays, which can be used for diagnostic visualisation of tumours, and delivering effective doses of β-particle radiation to kill cancer cells [146,154]. Lutetium-177 is one of the two only clinically usable radioisotopes that can simultaneously emit γ ray and β-particle radiation, making it a desirable option without needing to pair two different radioisotopes such as technetium-99 m (diagnostic) and rhenium-188 (therapeutic) [155,156]. The other radioisotope that also simultaneously emits β-particles and γ-rays is iodine-131 [157].

Previous research with a [^177^Lu]Lu-labelled HER2 antibody, trastuzumab, and a nanobody (2Rs15d), demonstrated successful targeting for in vivo imaging and tumour reduction using [^177^Lu]Lu-DPTA-2Rs15d in HER2-tumour-bearing mice [154]. Compared to [^177^Lu]Lu-DPTA-trastuzumab, which exhibited high levels of non-specific radiation accumulation in healthy tissues, blood, liver, spleen and kidneys, [^177^Lu]Lu-DPTA-2Rs15d demonstrated specific tumour targeting properties [154]. The only non-specific accumulation was observed in the kidneys due to renal clearance; however, no toxicity was observed [145].

Using radiolabelled gold nanoparticles conjugated to aptamers—[^177^Lu]Lu-Au-NLS-RGD-anti-VEGF—González-Ruíz et al. [158] demonstrated successful targeting in in vivo imaging and tumour reduction in mice bearing U87MG gliomas. Using Cerenkov luminescence imaging, which detects Cerenkov radiation (charged particles traveling faster than light in a medium) generated from beta particles of lutetium-177, this showed high activity associated with the tumour region, suggesting high radiopharmaceutical retention and uptake [158]. Compared to the liver, the spleen and kidneys had lower uptake (<0.7%). Uptake of [^177^Lu]Lu-Au-NLS-RGD-anti-VEGF was significantly greater in the tumour at 96 h post-injection (38.4%). Furthermore, targeted radiotherapy with [^177^Lu]Lu-Au-NLS-RGD-anti-VEGF significantly reduced tumour size in U87MG-glioma-bearing mice compared to the control (untreated) and thermotherapy (laser irradiation) after 25 days [158]. The tumour killing ability of the [^177^Lu]Lu-Au-NLS-RGD-anti-VEGF aptamer was shown to be synergistically enhanced when coupled with thermotherapy: the resultant tumour size was 28 times smaller than after monotherapy with laser irradiation and 12 times smaller than after targeted radiotherapy alone [158]. These results and the development of the [^177^Lu]Lu-Au-NLS-RGD-anti-VEGF aptamer suggest that this nano-system could improve cancer treatment outcomes.

Zhang et al. [159] truncated a previously generated aptamer, JHIT2, which binds specifically to HepG2 cells, and investigated its capabilities as a dual-modality probe. The truncated aptamer, JHIT2e, had similar binding specificity and affinity to the parent JHIT2 aptamer, and was radiolabelled with iodine-131 via the carboxyfluorescein (FAM) chelator [159]. Although Zhang et al. [159] did not investigate the efficacy of the [^131^I]I-FAM-JHIT2e aptamer in human hepatoma cancer cells, in vitro analysis confirmed that the [^131^I]I-FAM-JHIT2e aptamer maintained its binding specificity and radioactivity. Due to simultaneous emissions of both γ-rays and β-particles of iodine-131, this study provides proof-of-concept evidence for theranostic treatments with iodine-131, conjugated to aptamers, for highly targeted radiotherapy.

Radiolabelling aptamers with these unique radioisotopes may improve diagnosis and therapeutic treatments for BC patients. Transferring the radiolabelling processes with lutetium-177 or iodine-131 from mAbs and nanobody-based molecular agents to aptamers could potentially lead to improved clinical outcomes, given the desirable properties and advantages of aptamers over their protein-based predecessors [22,23,24,118,122].

## 10. Limitations

Given the broad spectrum of encouraging advantages over antibodies, clinical translation of aptamers for use in diagnostics and therapeutics are hindered by certain limitations [160,161]. The main limitations which challenge the applicability of aptamers include susceptibility to endogenous nuclease degradation, rapid renal clearance and the potential to initiate innate immune responses [161,162]. Such limitations may be overcome through chemical modification of aptamers to improve their pharmacological properties and binding affinities [120,161,163].

Due to the obvious nature of aptamers, being composed of DNA or RNA, they are susceptible to rapid nuclease hydrolysis in vivo, which limits their biological stability in the body [160,161,163]. To improve the nuclease resistance, capping the 3′-end with inverted deoxy-thymidine, which has a 3′–3′ linkage, can increase the stability of the aptamers against 3′-5′ exonuclease activity in human serum [161,163]. Gijs et al. [124] modified HER2 aptamers by capping the 3′-end with inverted deoxy-thymine and the 5′-end with maleimide-NOTA, and extended the aptamers’ biological half-life (184.6 min ± 21.5 min) when compared to their unmodified counterparts (121.9 min ± 45.1 min), indicating improved stability against nuclease-mediated degradation in plasma. Previously, biotin modification at the 3′-end was demonstrated to significantly improve 3′-exonuclease resistance in rodent blood serum and decrease the clearance rate of the aptamers in blood circulation in vivo [161,164].

Additionally, due to the smallness of aptamers, they are susceptible to rapid excretion by renal glomerular filtration [161]. Conjugation to polyethylene glycol (PEG), which has previously been demonstrated to improve drug plasma half-life and reduce degradation by metabolic enzymes, can also be utilised for aptamers [163]. Attachment of a high-molecular-weight PEG to an aptamer can decrease its rate of renal clearance and increase systemic circulation time by increasing its overall size [160,161,163]. However, repeated administrations of PEGylated compounds have been reported to induce anti-PEG antibodies [165]. As a result, due to pre-existing anti-PEG antibodies, adverse reactions were reported in human clinical trials that were evaluating the efficacy of a PEGylated RNA aptamer, pegnivacogin [166,167]. Additionally, a study by Moreno et al. [165] demonstrated anti-PEG antibodies can bind to and inhibit the therapeutic activities of an anticoagulant PEGylated RNA aptamer in vitro and in vivo, indicating that anti-PEG antibodies can compromise the therapeutic efficacy of aptamers. It is worth noting that the PEG-related reactions are not limited to aptamers, but are also observed in PEGylated drugs, proteins and nanoparticles [168]. Alternatively, an aptamer can be modified with a lipid moiety such as cholesterol to increase its molecular size, in order to extend its plasma half-life in systemic circulation and evade renal filtration and clearance [161,163]. In a study by Lee, et al. [169], the conjugation of cholesterol to a 2′-F pyrimidine-modified RNA aptamer targeting the non-structural protein 5B of hepatitis C virus, extended the plasma half-life, and accordingly reduced renal clearance by approximately nine-fold when compared to the non-conjugated aptamer.

Aptamers are considered inert and non-immunogenic due to the low immunogenicity of nucleic acids; however, certain conditions, such as the aforementioned PEGylation or conjugation to unmethylated dinucleotide sequence 2′-deoxycytidine-phosphate-2′-guanine (CpG), can activate the immune system [160,162,163,170]. The innate immune system can be activated due to the CpG sequences in DNA or RNA oligonucleotides, which can act like pathogen-associated molecular patterns (PAMPs) and trigger cytokine production via Toll-like receptors (TLRs) [162]. Therefore, therapeutic aptamers with CpG or CpG-containing segments can have deleterious side-effects in patients, if the sequences are not neutralised [162]. However, due to their easily modifiable nature, aptamers can be synthesised with corrective strategies to neutralise the PAMP-like effects of the CpG-containing segments, as long as the strategies do not alter the binding specificity or affinity [24,162]. Such approaches may include the methylation of cytosines in the aptamer or masking the CpG segment with a second moiety; truncation of the aptamer down to its simplest binding unit to excise the CpG segment; or backbone modification of the sugar moieties to lessen the immune response [162]. Alternatively, competitive agents can be utilised via the co-administration of another aptamer or TLR-suppressive drugs to antagonise the TLR and neutralise their damaging effects [162].

## 11. Conclusions

BC is a highly heterogenous disease, and the overexpression of HER2 is strongly associated with poorer prognosis. Due to a lack of symptoms and the current imaging modalities lacking the appropriate balance of specificity and sensitivity, there are delays in detecting and diagnosing BC at an early stage for curative surgical treatments.

Aptamers are single-stranded nucleic acids that are capable of recognising targets and binding to them with high specificity, selectivity and affinity. Aptamers are advantageous to current antibody-based therapeutics due to their increased pH and temperature stability, non-immunogenicity, low production cost and simple modification and functionalisation processes. The high specificity of aptamers, coupled with their ability to become functionalised with appropriate radioisotopes, could enable specific and sensitive nuclear medical imaging of tumours to better detect and diagnose BC at an earlier stage, and advance nuclear medicine in the battle against cancer.

We have described several aptamers that target the HER2 biomarker for the diagnosis and potential treatment of HER2(+) BC. The aptamers described provide promising and exciting avenues for future targeted therapeutics, when combined with radiopharmaceuticals, to further expand the area of personalised and targeted medicine. However, further research and effort are warranted to expand on the current knowledge of HER2 aptamers and establish effectiveness, aptamer–target interactions, safety and pharmacokinetics in vivo to enable existing HER2 aptamers to enter clinical trials. Previous in-vivo research provides strong proof-of-concept evidence for the use of aptamers in targeted therapeutics, however, rigorous in-vivo randomized controlled trials are needed to establish its effectiveness when compared to current anti-HER2 therapies. Further research needs to explore important factors, such as in vivo stability; demonstrating the target specificity of aptamers reaching sites of primary and secondary metastasis in vivo; and modifications of aptamers to increase circulation time and overcome renal excretion in vivo without compromising functionality. Ultimately, this will enable future translations into clinical practice as novel and primary treatments, or as adjuvant therapies to current conventional anti-HER2 therapeutics.

## Figures and Tables

**Table 1:** The different methods of tumour detection by current medical imaging modalities and their contrast agents or radionuclides—advantages and disadvantages. Adapted from [65,67,72,74,77,78,79,80,81,82,83,84].

Medical Imaging Tool	Contrast Agents/Radionuclides	Method of Detection	Advantages	Disadvantages
Ultrasound	Microbubbles	Measures blood flow and tissue perfusion	Non-nephrotoxic	Operator dependent
		Accumulates in the intravascular space	Non-ionising	
			Real time information	IV administration is invasive
			Well tolerated by patients	Unstable and early breakdown of microbubbles can limit examination time
X-Ray CT	Iodine	Uptake of iodine by tissues increases its linear attenuation coefficient	Fast acquisition time	Ionising radiation
				Large concentration of iodine
				Potential toxicity
		Attenuates x-ray beam	Greater sensitivity	Renal complications
		Localised iodine accumulation with malignant lesions to create greater image contrast	3-D image reconstruction	Possible adverse events of allergic reactions
MRI	Gadolinium Superparamagnetic iron oxide	CA’s accumulates in tumour	Non-ionising radiation	Free gadolinium ions are highly toxic (Nephrogenic Systemic Fibrosis)
			Multiplanar reformation	Free gadolinium can interfere with calcium and protein binding sites
			Images multiple intrinsic property of tissues	
		Gadolinium shortens T_1_ relaxation time and creates bright intensity (hyper-intense) contrast	Rapid renal excretion	
		Superparamagnetic iron oxide nanoparticles shortens T_2_ relaxation time and creates a negative (hypo-intense) image contrast	Superior image quality	
PET	Positron emitting radioisotopes: ^18^F, ^15^O, ^13^N, ^11^C	Uptake of radiotracer by tissues	Provides information on physiological activity	Lacks anatomical detail
				IV administration considered invasive
		Annihilation of positrons with surrounding electrons and produces two high energy gamma rays	Can provide early detection of tumours based on enhanced metabolism of tumour cells compared to normal tissues	Small tumours may be missed due to partial volume effect
		Gamma rays are detected by PET camera	High sensitivity	Non-specific uptake of radiotracer can occur in highly metabolically active tissues leading to false positives
		Tumour imaging relates to the differences in physiological and metabolic properties of normal tissues and tumours	Specificity can be increased by radiolabelling exogenous probes (pre-clinical)	Radiation exposure concerns
SPECT	Gamma emiting photons: ^99m^Tc, ^123^I, ^125^I	Uptake of radiotracer by tissues	Provides information on physiological activity	Lacks anatomical detail
				IV administration considered invasive
			Specificity can be increased by radiolabelling exogenous probes (pre-clinical)	Non-specific uptake of radiotracer can occur in highly metabolically active tissues leading to false positives
		Gamma photons are emitted and picked up by gamma cameras	Can reach greater resolutions than PET (less than 1mm)	limited number of photons due to maximum allowable dose of radiation that can be administered

**Table 2:** Different aptamers radiolabelled for molecular imaging investigations.

Aptamer	Radiolabel/Nanoprobe	Target	Indication	References
AS1411	^64^Cu	Nucleolin	Over-expressed on cell-surface of various cancers (breast, cervical, hepatocellular, lymphocytic leukemia, prostate, renal)	[125] [126] [127]
	Fe_3_O_4_@Au			
	MNP@SiO_2_(RITC)-PEG/COOH/pro-N/NH_2_			
Aptamer 2-2 HeA2_1 HeA2_3 SE15-8 Sk6Ea	^68^Ga	HER2	Over-expressed in various cancers (bladder, breast, gastric, lung, ovarian, salivary, stomach)	[124] [28] [27] [108]
	^18^F			
	FAM (Carboxyfluorescein)			
F3B	^111^In	Matrix metalloproteinases-9 (MMP-9)	Over-expressed in malignant melanoma	[128] [119]
	^99m^Tc			
A10	^64^Cu	Prostate cancer-specific cell-surface antigen (PSMA)	Transmembrane protein that is overexpressed in prostate cancer cells	[122]

**Table 3:** Summary of findings of current HER2 aptamers. Adapted from [27,28,46,123,124].

Aptamer	Predicted Structure	Binding Affinity	In Vitro/In Vivo Results
HB5	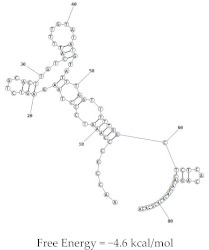	HER2 peptide: K_d_ = 18.9 nM	Not yet explored in vivo
		HER2 ECD: K_d_ = 316 nM	
HeA2_1	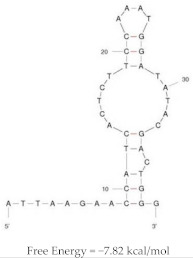	HER2 peptide: K_d_ = 28.9 nM	PET imaging with ^68^Ga-HeA2_1 in tumour bearing mice
			Mice inoculated with SKOV-3 or MDA-MB-231 cells
			Demonstrated stability in mouse plasma ex vivo
			Significant uptake and accumulation of aptamer in HER2 + SKOV-3 tumours, compared to MDA-MB-231 (1.5 fold higher)
			Rapid accumulation in liver, kidney and bladder reflects major clearance pathways
HeA2_3	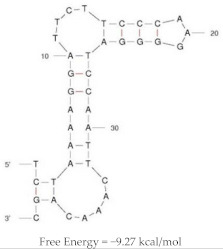	HER2 peptide: K_d_ = 6.2 nM	PET imaging with ^68^Ga-HeA2_3 in tumour bearing mice
			Mice inoculated with SKOV-3 or MDA-MB-231 cells
			Demonstrated stability in mouse plasma ex vivo
			Significant uptake of aptamer in HER2 + SKOV-3 tumours, compared to MDA-MB-231 (1.5 fold higher)
			Rapid accumulation in liver, kidney and bladder reflects major clearance pathways
SH-1194-35	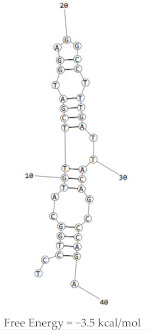	K_d_ values Not provided	PET imaging with ^18^F-SH-1194-35 in tumour bearing mice
			Mice inoculated with BT474 or MDA-MB-231 cells
			Significant uptake and accumulation of aptamer in HER2 + BT474 tumour bearing mice, compared to HER2- MDA-MB-231 tumours
			Biodistribution demonstrated high accumulation in two major excretory pathways: kidneys and intestine
Heraptamer1	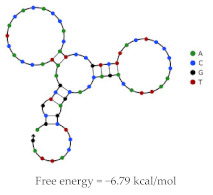	K_d_ = 5.1 ± 5.3 nM	Aptamer were characterised in vitro for binding affinity against HER2-ECD coupled beads and SKOV3 cells
			Aptamers were radiolabelled with ^18^F for in vivo imaging of tumour bearing mice
			Mice inoculated with SKOV3 or MDA-MB-231 cells
			Significant uptake and accumulation of aptamer in HER2 + SKOV3 tumour bearing mice, compared to HER2-MDA-MB-231 tumours
			High accumulation of radioactivity was noted in bladders and kidneys, reflecting renal excretion
			Accumulation of radioactivity was noted in gallbladder, indicating aptamer metabolism
Heraptamer2	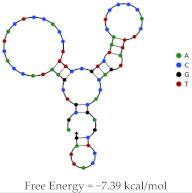	K_d_ = 23.7 ± 11.2	Aptamer were characterised in vitro for binding affinity against HER2-ECD coupled beads and SKOV3 cells
			Aptamers were radiolabelled with ^18^F for in vivo imaging of tumour bearing mice
			Mice inoculated with SKOV3 or MDA-MB-231 cells
			Significant uptake and accumulation of aptamer in HER2 + SKOV3 tumour bearing mice, compared to HER2-MDA-MB-231 tumours
			High accumulation of radioactivity was noted in bladders and kidneys, reflecting renal excretion
			Accumulation of radioactivity was noted in gallbladder, indicating aptamer metabolism
